# Cord Blood Mercury and Early Child Development: Lederman and Perera Respond

**DOI:** 10.1289/ehp.0800155R

**Published:** 2009-01

**Authors:** Sally Ann Lederman, Frederica P. Perera

**Affiliations:** Columbia Center for Children’s Environmental Health, Columbia University, New York, New York, E-mail: sal1@columbia.edu

In our study (Lederman et al. 2008), we examined the relation of cord and maternal blood mercury levels to child developmental outcomes at 1, 2, 3, and 4 years of age in a cohort whose mothers were selected because they were pregnant on 11 September 2001. Some of the women were exposed to the World Trade Center (WTC) event because they lived and/or worked near the WTC site in the weeks after the disaster, whereas others lived and worked elsewhere (reference group).

In his letter, Dórea raises two issues related to our finding that cord blood Hg across all groups was associated with reduced cognitive function at 3 and 4 years of age. The first is a concern that either maternal exposure during pregnancy or infant post-natal exposure to ethyl mercury (from vaccines containing the preservative thimerosal) may have influenced the neurodevelopmental scores that we measured in our study (Lederman et al. 2008). Dórea indicates, however, that this preservative was removed from most infant vaccines around the time of the WTC disaster; this is supported by data from the Food and Drug Administration (2005).

With regard to maternal exposure to Hg from vaccines (infant prenatal exposure), we measured total blood Hg. Therefore, our blood Hg levels included Hg from that source, and would have then contributed to the effects we reported to be associated with blood Hg levels. Regarding postnatal Hg exposure, our 329 subjects were enrolled at delivery between December 2001 and June 2002. Seven women were enrolled in December 2001, and each provided a cord blood sample. Thus, with regard to later vaccinations, our cohort had a low risk of post-natal thimerosal exposure. Moreover, if such vaccine exposure had occurred, we would expect that all of our study children would have had a similar chance of exposure, and that postnatal thimerosal exposure would be unrelated to prenatal Hg levels (limiting the possibility that this exposure confounds the relationship between cord blood Hg and cognitive development). However, such postnatal Hg exposure would have increased the variability of measured cognitive function at any given level of cord blood Hg, which would have reduced our ability to distinguish cognitive decrements related to prenatal Hg exposure.

The second issue raised by Dórea relates to the potential effect of infant feeding method on cognitive function. He is not correct that we left the mode of feeding out of the model relating infant Hg exposure to cognitive development. As shown in our Table 5 (Lederman et al. 2008), full models controlled for breast-feeding, using a variable that combined the weighted durations of exclusive breast-feeding and mixed feeding. We also controlled for race, maternal IQ, per capita family income, maternal age, environmental tobacco smoke exposure during pregnancy, marital status, education, material hardship, and the child’s sex, gestational age at birth, and exact age in days at testing. As indicated in the “Results” of our article (Lederman et al. 2008), the reduced models in Table 5 excluded variables with *p* > 0.1. Our measure of breast-feeding was excluded from the reduced models on this basis.

We conclude that neither vaccine exposure to Hg nor breast-feeding status had important effects on the developmental outcomes we studied in the WTC cohort (Lederman et al. 2008) or their relation to prenatal Hg exposure.
